# Association of 25-hydroxyvitamin D levels with lipid profiles in osteoporosis patients: a retrospective cross-sectional study

**DOI:** 10.1186/s13018-023-04079-8

**Published:** 2023-08-14

**Authors:** Si-ming Xu, Ke Lu, Xu-feng Yang, Yao-wei Ye, Min-zhe Xu, Qin Shi, Ya-qin Gong, Chong Li

**Affiliations:** 1 Department of Orthopedics, Gusu School, Nanjing Medical University, The First People’s Hospital of Kunshan, Suzhou, 215300 Jiangsu China; 2 Department of Orthopedics, Affiliated Kunshan Hospital of Jiangsu University, No. 566 East of Qianjin Road, Suzhou, 215300 Jiangsu China; 3 Department of Orthopedics, The First Affiliated Hospital of Soochow University, Orthopedic Institute of Soochow University, Suzhou, 215031 Jiangsu China; 4 Information Department, Affiliated Kunshan Hospital of Jiangsu University, Suzhou, 215300 Jiangsu China

**Keywords:** 25(OH)D, Blood lipid, Osteoporosis, HDL-C, LDL-C, TG, TC

## Abstract

**Background:**

In the literature, scarce data investigate the link between 25-hydroxyvitamin D (25[OH]D) and blood lipids in the osteoporosis (OP) population. 25(OH)D, as a calcium-regulating hormone, can inhibit the rise of parathyroid hormone, increase bone mineralization to prevent bone loss, enhance muscle strength, improve balance, and prevent falls in the elderly. This retrospective cross-sectional study aimed to investigate the association between serum 25(OH)D levels and lipid profiles in patients with osteoporosis, with the objective of providing insight for appropriate vitamin D supplementation in clinical settings to potentially reduce the incidence of cardiovascular disease, which is known to be a major health concern for individuals with osteoporosis.

**Methods:**

This is a retrospective cross-sectional study from the Affiliated Kunshan Hospital of Jiangsu University, including 2063 OP patients who received biochemical blood analysis of lipids during hospitalization from January 2015 to March 2022. The associations between serum lipids and 25(OH)D levels were examined by multiple linear regression. The dependent variables in the analysis were the concentrations of serum lipoprotein, total cholesterol (TC), triglycerides (TGs), apolipoprotein-A, lipoprotein A, high-density lipoprotein cholesterol and low-density lipoprotein cholesterol (LDL-C). The independent variable was the concentration of blood serum 25(OH)D. At the same time, age, body mass index, sex, time and year of serum analysis, primary diagnosis, hypertension, diabetes, statins usage, beta-C-terminal telopeptide of type I collagen, procollagen type I N-terminal propeptide were covariates. Blood samples were collected in the early morning after the overnight fasting and were analyzed using an automated electrochemiluminescence immunoassay on the LABOSPECT 008AS platform (Hitachi Hi-Tech Co., Ltd., Tokyo, Japan). The generalized additive model was further applied for nonlinear associations. The inception result for smoothing the curve was evaluated by two-piecewise linear regression exemplary.

**Results:**

Our results proved that in the OP patients, the serum 25(OH)D levels were inversely connected with blood TGs concentration, whereas they were positively associated with the HDL, apolipoprotein-A, and lipoprotein A levels. In the meantime, this research also found a nonlinear relationship and threshold effect between serum 25(OH)D and TC, LDL-C. Furthermore, there were positive correlations between the blood serum 25(OH)D levels and the levels of TC and LDL-C when 25(OH)D concentrations ranged from 0 to 10.04 ng/mL. However, this relationship was not present when 25(OH)D levels were higher than 10.04 ng/mL.

**Conclusions:**

Our results demonstrated an independent relationship between blood lipids and vitamin D levels in osteoporosis patients. While we cannot establish a causal relationship between the two, our findings suggest that vitamin D may have beneficial effects on both bone health and blood lipid levels, providing a reference for improved protection against cardiovascular disease in this population. Further research, particularly interventional studies, is needed to confirm these associations and investigate their underlying mechanisms.

**Supplementary Information:**

The online version contains supplementary material available at 10.1186/s13018-023-04079-8.

## Introduction

Osteoporosis (OP) represents a skeletal sickness described by weakened bone mass and bone microarchitecture that quickly leads to bone fractures [1]. OP diagnosis is based on bone mineral density (BMD) [1]. Data show that in China in 2019, the estimated age‐standardized occurrence of OP at the backbone or hip in males and females 50 years old and above was 6.46% and 29.13%, respectively [2]. Therefore, 10.9 million Chinese males and 49.3 million females were projected to have OP. Certain OP risk factors were further recognized, such as the way of living, food regimes, comorbid diseases and medications, and genetic predispositions [2, 3]. Recent data linked OP patients' metabolism and blood serum lipid profile with the disease [4–6]. It has been shown that vitamin D led to an increase in BMD in OP individuals [7], whereas the lipid profile was reported as a risk factor for OP [8, 9].

Vitamin D is a critical steroid-like vitamin for human health and is produced in humans by irradiation with ultraviolet radiation B (UVB) light [10]. In the liver, it metabolizes to calcifediol [25(OH)D] and then to 1.25-dihydroxy vitamin D [1.25(OH)D] in the kidneys. 1.25(OH)D is a transcription factor. It regulates the activity of more than 1000 different genes by binding to vitamin D receptors (VDRs) [11]. Recent data appointed the blood serum concentration of calcifediol as a medical indicator for assessing vitamin D metabolism and absorption in the body [12, 13]. Vitamin D affects the total mineralization of the bones, the rate of bone resorption and the incidence of bone breaks. Epidemiological investigations display the link between the shortage of it with low bone thickness, higher bone resorption and higher breakage occurrence. Therefore, additional uptake of this vitamin results in elevated BMD, a reduction in bone resorption and a drop in fracture occurrence [14]. On the other side, a meta-analysis of 41 RCTs evaluated that the benefits of vitamin D for lipid metabolism are well-known [15, 16].

Data show that medical conditions with abrogated lipid metabolism, also known as dyslipidemias, are the lead cause of a wide range of cardiological complications such as atherosclerotic cardiovascular diseases (ASCVD), among which the most common is coronary heart disease (CHD) [17]. OP and CHD share typical age-associated onset and fundamental pathogenetic mechanisms such as bone and vascular mineralization [18]. Furthermore, data show a link between the concentration of blood serum cholesterols [total cholesterol (TC), triglycerides (TGs), high-density (HDL-C), and low-density lipoprotein cholesterol (LDL-C)] and BMD [4, 5], thus proving that vitamin D is intricately related to the cholesterol metabolism biosynthesis pathway. This interplay is multifaceted.

Moreover, vitamin D shortage has been related to the augmented occurrence of cardiovascular diseases (CVD) [19, 20]. Some data further show that statin therapy, used in the management of hypercholesterolemia, does not affect the plasma levels of this vitamin [21]. Moreover, data confirm that vitamin D and cholesterol dysregulation are age-associated [22, 23]. However, there is little knowledge about the relationship between the two in OP patients.

Here, this research proves the independent association between the serum cholesterol levels (TC, HDL-C, TG, and LDL-C) in males and females, age ≥ 50 years, with 25(OH)D blood serum concentration in Chinese patients with OP.

## Materials and methods

### Study design and patients' data

**Fig. 1:**
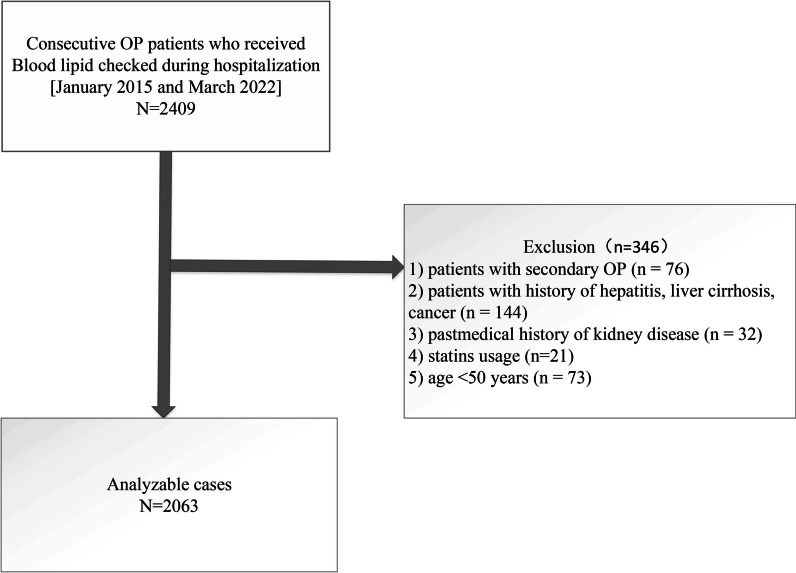
Study flow chart

This study has performed a retrospective investigation, which included patients' data collected between January 2015 and March 2022. The patients' medical data were retrieved from the Affiliated Kunshan Hospital of Jiangsu University, Suzhou, China. 2409 OP patients were included in the study. All of them received medical blood checks during hospitalization. OP diagnosis was made on the following inclusion criteria: (1) occurrence of bone instability and breaks in the lack of other metabolic bone illnesses, with physiological BMD (T-score), and (2) OP confirmed based on a T-score of − 2.5 or less, even in the lack of a predominant bone rupture [7]. The exclusion criteria were: (1) patients with secondary OP (*n* = 76), or (2) patients with a history of hepatitis, liver cirrhosis, or cancer (*n* = 144); (3) medical history of kidney disease (*n* = 32), and (4) statins usage (*n* = 21); (5) age < 50 years (*n* = 73). After applying the inclusion criteria, 2063 patients were acquired for the study. Figure 1 represents patients' data and medical history. The study was approved by the Ethics Committee at the Affiliated Kunshan Hospital of Jiangsu University, Suzhou, China (approval No. 2020-03-046-K01) and was compliant with the Declaration of Helsinki. The patients' identity was hidden for an unbiased investigation. All patients signed a written consent form.

### Dependent variables

The blood analyses were done on early morning fasting blood, and the lipids' quantitation was performed with an automated electro-chemiluminescence immunoassay on the LABOSPECT 008AS platform (Hitachi High-Tech Co., Tokyo, Japan). In our study, the dependent variables were the serum concentrations of TC, LDL-C, TG, apolipoprotein-A (APO-A), HDL-C, and lipoprotein A (LPA).

### Exposure variables

Our study's exposure variable was 25(OH)D in its superabundant serum form, 25(OH)D. An automatic electro-chemiluminescence immune assay measured its concentrations through the Roche Cobas 8000/e602 analyzer (Roche Diagnostics, Mannheim, Germany). The blood collection moment was included in the analysis, together with the seasons.

### Covariates

Covariates were age, sex, BMI, season and year of blood collection, primary diagnosis (OP without fractures/OPF), hypertension, diabetes, statins usage, beta-C-terminal telopeptide of type I collagen(β-CTX), and procollagen type I N-terminal propeptide (P1NP). All clinical variables were quantified within 3 days of hospital admittance.

### Statistics

All the results are presented as mean ± standard deviation (SD), as a median (Q1 Q3) and as frequency (%) for constant and categorical variables, respectively. Pearson's chi-square or Fisher's exact tests were applied for univariate data quantitating absolute values. The *t* test and the Mann–Whitney U test were applied for continuous variables with standard and non-normally distributed continuous data, respectively. The univariate logistic regression data quantitation was utilized for assessing the link between the serum 25(OH)D levels and blood lipids.

The generalized estimating equations [24] studied the independent relations between the blood lipids' concentrations and the serum 25(OH)D by controlling the influence of covariances. Data were quantified through unadjusted (basic), negligibly accustomed (Model I) or fully adjusted (Model II) models. The variance inflation factor (VIF) data evaluation allowed adjustment of covariances following criteria: (1) the covariate was included in the crude model or detached from the full one, while the similar odds ratio (OR) was altered by at least 10%; (2) when the covariate from criterion 1 had *P* value of < 0.1 in the univariate model [22]. So, in terms of fully adjusted models, Model II was developed based on Criteria 1.

The generalized additive model (GAM) identified nonlinear relationships among our data. The two-piecewise linear regression model sets the threshold for line smoothing. The recursive method was used to spontaneously evaluate the turning point in the case of an apparent ratio in the smoothing curve [23]. Furthermore, this research performed subgroup analyses and estimated their robustness and potential variations, stratifying different covariates. Finally, the modifications and interactions of the subgroups were analyzed using the likelihood ratio test (LRT).

To enhance the generalizability of the findings for future studies, a sensitivity analysis was performed to examine the impact of BMI classification. Specifically, BMI was classified according to both the WHO international classification [25] and the classification used in the Chinese adult population [26]. The sensitivity analysis was conducted with the aim of determining whether the results were affected by the selected BMI classification system and to ensure that the findings could be applied to diverse populations beyond China.

All statistical analyses were performed using the Empower Stats (www.empowerstats.com, X&Y Solutions, Inc., Boston, MA, USA). The R software version 3.6.3 was also applied (http://www.r-project.org). *P*-values less than 0.05 were accepted as statistically significant.

## Results

### Features of study contributors

A population-descriptive analysis was performed to characterize the OP population included in the study. A total of 2063 patients, 83.86% (*n *= 1730), average age = 68.5 years, were females who passed the inclusion criteria and enrolled in this retrospective investigation. The medical data are included in Table 1. The blood serum 25(OH)D levels were measured, and the mean (SD) and medium (Q1–Q3) concentrations were 21.13 (8.83) ng/mL and 20.00 (15.00–25.83) ng/mL, respectively. The patients diagnosed with OPF accounted for 32.574% (*n* = 672). The mean and median concentrations of serum TC were 4.564 (0.997) ng/mL and 4.525 (3.890–5.160) ng/mL, respectively. These values for TG were 1.361 (0.876) ng/mL and 1.130 (0.820–1.647) ng/mL, individually, whereas for HDL concentrations were 1.467 (0.331) ng/mL and 1.430 (1.230–1.660) ng/mL, correspondingly. LDL-C mean (SD) and median (Q1-Q3) concentrations were 2.713 (0.787) ng/mL and 2.660 (2.160–3.210) ng/mL and of APO-A were 1.368 (0.274) ng/mL and 1.340 (1.180–1.530) ng/mL. For the blood serum concentrations these values were 186.168 (166.013) ng/mL and 131.000 (73.250–243.000) ng/mL, respectively, whereas for HCY were 12.968 (6.373) ng/mL and 11.360 (9.270–15.040) ng/mL. The concentrations of serum Ca, β-CTx and P1NP were correspondingly measured. This research classified the patients into five groups based on the estimated BMI (kg/m_2_). The groups were the following (1) ≤ 18.5 (underweight); (2) > 18.5 and ≤ 23.9 (normal); (3) > 23.9 and ≤ 28 (overweight); (4) > 28 and ≤ 35 (obesity); (5) > 35 (severe obesity). The BMI for the study participants was normal at 60.834% (*n* = 1255). The variables like season and year of blood sampling were measured too. The seasons of blood sampling were spring, summer, autumn and winter, whereas the years of blood collection were distinguished from 2015 to 2022. 31.168% (*n* = 643) of the study participants had hypertension, while 14.106% (*n* = 291) had diabetes.

**Table 1:** Characteristics of study participants

Variables	(*N*) Mean (SD) Median (Q1–Q3)^a^
25(OH)D level continuous, ng/mL	(2063) 21.13 (8.83) 20.00 (15.00–25.83)
TC, mmol/L	(1566) 4.56 (1.00) 4.53 (3.89–5.16)
TG, mmol/L	(1566) 1.36 (0.88) 1.13 (0.82–1.65)
LDL, mmol/L	(1566) 2.71 (0.79) 2.66 (2.16–3.21)
HDL, mmol/L	(1566) 1.47 (0.33) 1.43 (1.23–1.66)
APO-A, g/L	(1566) 1.37 (0.27) 1.34 (1.18–1.53)
APO-B, g/L	(1566) 0.89 (0.23) 0.88 (0.73–1.04)
LPa, mg/L	(1566) 186.17 (166.01) 131.00 (73.25–243.00)
HCY, umol/L	(1567) 12.97 (6.37) 11.36 (9.27–15.04)
Ca, mmol/L	(2029) 2.26 (0.17) 2.26 (2.17–2.35)
β-CTX, ng/mL	(2045) 0.42 (0.30) 0.34 (0.19–0.59)
P1NP, ug/L	(2044) 50.44 (34.64) 43.00 (29.00–64.00)
Sex, *N* (%)	
Male	333 (16.14%)
Female	1730 (83.86%)
Age (years)	(2063) 68.51 (8.66) 68.00 (63.00–74.00)
Age (years), *N* (%)	
> 50, ≤ 70	1256 (60.88%)
> 70	807 (39.12%)
BMI (kg/m^2^), *N* (%)	
≤ 18.5	99 (4.80%)
> 18.5, ≤ 23.9	1255 (60.83%)
> 23.9, ≤ 28	556 (26.95%)
> 28, ≤ 35	147 (7.13%)
> 35	6 (0.29%)
Season of blood collection, *N* (%)	
Spring (March, April and May)	503 (24.38%)
Summer (June, July and August)	509 (24.67%)
Autumn (September, October and November)	608 (29.47%)
Winter (December, January and February)	443 (21.47%)
Year of blood collection, *N* (%)	
2015	21 (1.02%)
2016	32 (1.55%)
2017	48 (2.33%)
2018	128 (6.20%)
2019	458 (22.20%)
2020	578 (28.02%)
2021	705 (34.17%)
2022	93 (4.51%)
Hypertension, *N* (%)	
No	1420 (68.83%)
Yes	643 (31.17%)
Diabetes, *N* (%)	
No	1772 (85.89%)
Yes	291 (14.11%)
Main diagnosis, *N* (%)	
OP without fractures	1391 (67.43%)
OPF	672 (32.57%)

*SD* standard deviation, *Q1* first quartile, *Q3* third quartile, *CI* confidence interval, *25(OH)D* 25-hydroxy vitamin D, *TC* total cholesterol, *TG* triglyceride, *HDL* high-density lipoprotein, *LDL* low-density lipoprotein, *APO-A* apolipoproteina, *APO-B* apolipoproteinb, *LPa* lipoproteina, *HCY* homocysteine, *Ca* calcium, *BMI* body mass index, *β-CTX* beta-C-terminal telopeptide of type I collagen, *P1NP* procollagen type I N-terminal propeptide, *OP* osteoporosis, *OPF* osteoporotic fracture

^a^For continuous variables

### Univariate analysis of blood lipids

The results of the univariate logistic regression analysis are displayed in Table 2. This research has revealed that the blood serum concentrations of 25OH(D) were negatively related to the concentrations of TG (β, -0.005; 95% CI [confidence interval], − 0.010, − 0.000; *P* = 0.04553), and positively associated with HDL (*β*, 0.005; 95% CI [confidence interval], 0.003, 0.006; *P* < 0.00001) and APO-A (*β*, 0.004; 95% CI [confidence interval], 0.003, 0.006; *P* < 0.00001).

**Table 2:** Univariate analysis for blood lipid

	Statistics (mean ± SD)^a^	TC β^b^ (95%CI) Pvalue	TG *β* (95% CI) *P* value	HDL *β* (95% CI) *P* value	LDL *β* (95% CI) *P* value	APO-A *β* (95% CI) *P* value	LPa *β* (95% CI) *P* value	HCY *β* (95% CI) *P* value
25(OH)D level continuous, ng/mL	21.13 ± 8.83	**0.003 (− 0.002, 0.009)** 0.25	**− 0.005 (− 0.010, − 0.000)** 0.05	**0.005 (0.003, 0.006)** **< 0.01**	**− 0.001(− 0.005, 0.004)** 0.78	**0.004 (0.003, 0.006)** < 0.01	0.80 (− 0.13, 1.74) 0.09	0.01 (− 0.02,0.05) 0.49
Sex, *N* (%)								
Male	333 (16.14%)	Reference	Reference	Reference	Reference	Reference	Reference	Reference
Female	1730 (83.86%)	0.54 (0.41, 0.68) < 0.01	0.25 (0.13, 0.36) 0.01	0.14 (0.09, 0.18) < 0.01	0.31 (0.20, 0.41) < 0.01	0.14 (0.11, 0.18) < 0.01	12.84 (− 9.53, 35.21) 0.26	− 3.12 (− 4.03, − 2.34) < 0.01
Age, *N* (%)								
> 50, ≤ 70	1256 (60.83%)	Reference	Reference	Reference	Reference	Reference	Reference	Reference
> 70	807 (39.12%)	− 0.37 (− 0.47, − 0.27) < 0.01	− 0.26 (− 0.34, − 0.17) < 0.01	− 0.03 (− 0.06, 0.005) 0.09	− 0.31(− 0.37, − 0.23) < 0.01	− 0.06 (− 0.09, − 0.03) < 0.01	6.94 (− 9.86, 23.73) 0.42	2.46 (1.83, 3.09) < 0.01
BMI, kg/m^2^, *N* (%)								
≤ 18.5	99 (4.80%)	Reference	Reference	Reference	Reference	Reference	Reference	Reference
> 18.5, ≤ 23.9	1255 (60.83%)	0.05 (− 0.20, 0.29) 0.71	0.49 (0.28 0.70) < 0.01	− 0.19 (− 0.27, − 0.11) < 0.01	0.24 (0.05, 0.43) 0.02	− 0.07 (− 0.14, − 0.005) 0.04	− 9.66(− 50.01, 30.69) 0.64	− 1.29 (− 2.84, 0.26) 0.10
> 23.9, ≤ 28	556 (26.95%)	0.09 (− 0.16, 0.34) 0.49	0.65 (0.43, 0.87) < 0.01	− 0.24 (− 0.32, − 0.15) < 0.01	0.33 (0.13, 0.53) < 0.01	− 0.05 (− 0.12, 0.02) 0.13	− 17.50 (− 59.46, 24.45) 0.41	− 1.64 (− 3.24, − 0.03) 0.05
> 28, ≤= 35	147 (7.13%)	0.12 (− 0.17, 0.42) 0.42	0.70 (0.45, 0.96) < 0.01	− 0.26 (− 0.36, − 0.16) < 0.01	0.41 (0.17, 0.62) < 0.01	− 0.06 (− 0.14, 0.02) 0.16	− 13.93 (− 63.14, 35.29) 0.58	− 0.48 (− 2.37, 1.41) 0.62
> 35	6 (0.29%)	− 0.34 (− 1.34, 0.67) 0.51	0.29 (− 0.58, 1.16) 0.52	− 0.18 (− 0.51, 0.16) 0.30	− 0.15 (− 0.94, 0.64) 0.71	0.04 (− 0.24, 0.31) 0.79	− 16.65 (− 184.09, 150.79) 0.85	− 3.32 (− 9.74,3.10) 0.31
Season of blood collection, *N* (%)								
Spring (March, April and May)	503 (24.38%)	Reference	Reference	Reference	Reference	Reference	Reference	Reference
Summer (June, July and August)	509 (24.67%)	− 0.02 (− 0.16, 0.12) 0.74	0.13 (0.003, 0.24) 0.04	− 0.05 (− 0.10, − 0.003) 0.04	− 0.09(− 0.20,0.02) 0.11	− 0.04 (− 0.08, − 0.004) 0.03	− 12.47(− 35.58, 10.64) 0.29	2.37 (1.49, 3.25) < 0.01
Autumn (September, October and November)	608 (29.47%)	0.05 (− 0.09 0.19) 0.47	0.03 (− 0.09, 0.15) 0.66	− 0.03 (− 0.07, 0.02) 0.20	− 0.04(− 0.15,0.07) 0.46	0.07 (0.04, 0.11) < 0.01	7.19 (− 15.31, 29.69) 0.53	1.38 (0.53, 2.24) < 0.01
Winter (December, January and February)	443 (21.47%)	0.01 (− 0.14, 0.15) 0.93	− 0.07 (− 0.20, 0.06) 0.31	0.04 (− 0.01, 0.09) 0.09	− 0.15(− 0.27, − 0.04) < 0.01	0.08 (0.04, 0.12) < 0.01	13.98(− 10.59,38.55) 0.26	0.53 (− 0.41, 1.46) 0.27
Ca, mmol/L	2.26 ± 0.17	1.01 (0.72, 1.30) < 0.01	0.37 (0.11, 0.63) < 0.01	0.22 (0.12, 0.32) 0.01	0.66 (0.43, 0.89) < 0.01	0.36 (0.28, 0.44) < 0.01	− 10.42(− 59.30,38.46) 0.68	− 3.27 (− 5.15, − 1.39) < 0.01
Hypertension, *N* (%)								
No	1420 (68.83%)	Reference	Reference	Reference	Reference	Reference	Reference	Reference
Yes	643 (31.17%)	− 0.28 (− 0.38, − 0.18) < 0.01	0.05 (− 0.04, 0.14) 0.27	− 0.09 (− 0.12, − 0.05) < 0.01	− 0.16 (− 0.24, − 0.08) < 0.01	− 0.05 (− 0.08, − 0.02) < 0.01	− 5.77 (− 22.94, 11.40) 0.51	− 0.31 (− 0.96, 0.35) 0.36
Diabetes, *N* (%)								
No	1772 (85.89%)	Reference	Reference	Reference	Reference	Reference	Reference	Reference
Yes	291 (14.11%)	− 0.39 (− 0.52, − 0.26) < 0.01	0.09 (− 0.02, 0.21) 0.10	− 0.10 (− 0.15, − 0.06) < 0.01	− 0.26(− 0.36, − 0.15) < 0.01	− 0.05 (− 0.08, − 0.01) 0.01	2.40 (− 19.50, 24.31) 0.83	− 1.32 (− 2.16, − 0.49) < 0.01
Year of blood collection, *N* (%)								
2015	21 (1.02%)	Reference	Reference	Reference	Reference	Reference	Reference	Reference
2016	32 (1.55%)	− 0.32 (− 0.88, 0.25) 0.27	− 0.27 (− 0.77, 0.22) 0.28	0.14 (− 0.04, 0.38) 0.13	− 0.32(− 0.76,0.12) 0.15	0.06(− 0.10, 0.21) 0.46	− 30.87(− 123.09,61.35) 0.51	− 0.90 (− 4.24, 2.44) 0.60
2017	48 (2.33%)	− 0.26 (− 0.78, 0.27) 0.34	− 0.16 (− 0.62, 0.29) 0.49	0.29 (0.12, 0.46) < 0.01	− 0.20(− 0.61,0.21) 0.34	0.19 (0.05, 0.34) < 0.01	− 7.30(− 93.42, 78.82) 0.87	0.37 (− 2.75, 3.49) 0.82
2018	128 (6.21%)	− 0.11 (− 0.58, 0.36) 0.65	− 0.20 (− 0.62, 0.21) 0.34	0.22 (0.06, 0.38) < 0.01	− 0.15 (− 0.52,0.22) 0.43	0.25 (0.12, 0.37) < 0.01	3.47 (− 74.05, 80.99) 0.93	1.09 (− 1.72, 3.90) 0.45
2019	458 (22.20%)	− 0.09 (− 0.54, 0.36) 0.70	− 0.18 (− 0.57, 0.22) 0.39	0.26 (0.11, 0.40) < 0.01	− 0.13(− 0.48,0.23) 0.48	0.19 (0.07, 0.32) < 0.01	19.96(− 54.06,93.99) 0.60	2.16 (− 0.52, 4.85) 0.11
2020	578 (28.02%)	− 0.15 (− 0.59, 0.30) 0.52	− 0.23 (− 0.62, 0.16) 0.25	0.28 (0.13, 0.43) < 0.01	− 0.08 (− 0.43,0.28) 0.68	0.25 (0.13, 0.37) < 0.01	− 19.75 (− 93.22,53.72) 0.60	3.71 (1.05, 6.37) < 0.01
2021	705 (34.17%)	− 0.13 (− 0.58, 0.32) 0.57	− 0.21 (− 0.61, 0.18) 0.29	0.20 (0.05, 0.35) < 0.01	− 0.22 (− 0.57,0.13) 0.23	0.21 (0.09, 0.33) < 0.01	− 53.14(− 126.46,20.17) 0.16	− 1.83 (− 4.48, 0.83) 0.18
2022	93 (4.51%)	− 0.26 (− 0.77, 0.24) 0.30	− 0.31 (− 0.75, 0.14) 0.17	0.21 (0.05, 0.38) 0.01	− 0.23 (− 0.63,0.17) 0.26	0.10 (− 0.04, 0.24) 0.16	5.09 (− 78.11, 88.28) 0.90	− 2.20 (− 5.21, 0.82) 0.15
Main diagnosis, *N* (%)								
OP without fractures	1391 (67.43%)	Reference	Reference	Reference	Reference	Reference	Reference	Reference
OPF	672 (32.57%)	− 0.14 (− 0.25, − 0.03) 0.02	− 0.08 (− 0.18, 0.02) 0.09	− 0.05 (− 0.09, − 0.02) < 0.01	− 0.05 (− 0.13,0.04) 0.32	− 0.07 (− 0.10, − 0.04) < 0.01	20.94 (2.18, 39.69) 0.03	1.03 (0.31, 1.75) < 0.01
β-CTX, ng/mL	0.42 ± 0.30	− 0.18 (− 0.35, − 0.01) 0.04	− 0.13 (− 0.27, 0.02) 0.10	− 0.13 (− 0.19, − 0.07) < 0.01	− 0.06 (− 0.19,0.08) 0.42	− 0.14 (− 0.19, − 0.09) < 0.01	33.14 (5.02, 61.25) 0.02	1.65 (0.57, 2.74) < 0.01
P1NP, ug/L	50.44 ± 34.64	− 0.003 (− 0.004, − 0.001) < 0.01	− 0.000 (− 0.002, 0.001) 0.54	− 0.001 (− 0.002, − 0.001) < 0.01	− 0.001(− 0.002, − 0.000) 0.03	− 0.001 (− 0.002, − 0.001) < 0.01	0.13 (− 0.10, 0.35) 0.27	0.02 (0.01, 0.03) < 0.01

Bolded data are now presented with three decimal places, which improves the sensitivity of the data

*SD* standard deviation, *CI* confidence interval, *25(OH)D* 25-hydroxy vitamin D, *TC* total cholesterol, *TG* triglyceride, *HDL* high-density lipoprotein, *LDL* low-density lipoprotein, *APO-A* apolipoproteina, *LPa* lipoproteina, *HCY* homocysteine, *Ca* calcium, *BMI* body mass index, *β-CTX* beta-C-terminal telopeptide of type I collagen, *P1NP* procollagen type I N-terminal propeptide, *OP* osteoporosis, *OPF* osteoporotic fracture

^a^For continuous variables

^b^The dependent variable was lipids and *β* is the result of univariate analysis for lipids level

In the univariate data quantitation of covariates like blood lipids and serum Ca_2_^+^ concentrations, the results showed a significant positive association with the concentrations of TC (*β*, 1.006; 95% CI, 0.715, 1.296; *P* < 0.00001), TG (*β*, 0.367; 95% CI, 0.108, 0.626; *P* value = 0.00548), HDL (*β*, 0.218; 95% CI, 0.121, 0.316; *P* = 0.00001), LDL-C (*β*, 0.663; 95% CI, 0.433, 0.893; *P* < 0.00001), APO-A (*β*, 0.357; 95% CI, 0.278, 0.436; *P* < 0.00001). Furthermore, this research detected a significant negative association with the levels of blood serum HCY (*β*, − 3.271; 95% CI, − 5.150, − 1.391; *P* = 0.00066). Females appeared with 54%, 24% and 31%greater TC, TG and LDL-C levels compared to males (*P* < 0.00001) and 13% higher LDL-C levels compared to men (*P* < 0.00001). OPF patients exhibited lower blood lipid levels compared with OP patients without fractures.

### Independent relation between the blood serum 25(OH)D concentrations and blood lipid profiles of patients

Multiple regression equations were used to analyze serum 25(OH)D and blood lipids to exclude the effect of confounding factors on the correlation between the two factors. Table 3 summarizes the independent link between serum 25(OH)D level and blood lipids using multivariate linear regression analysis. This research employed a two-level adjustment based on the covariance analysis applying the following models: the crude unadjusted, the one accustomed for sex, patients' years at the time of blood collection, time and season of sampling, the serum concentration of calcium, patients' BMI, primary diagnosis and Model II in sync to Model I including comorbidities like hypertension, diabetes, β-CTX and P1NP. The results demonstrated a major adverse relationship between TG and serum 25(OH)D levels in both the crude (*β*, − 0.005; 95% CI, − 0.01 to 0; *P* = 0.04553) and Model II (*β*, − 0.006; 95% CI, − 0.011 to − 0.001; *P* = 0.02954). These results showed that a 10 ng/mL increase in the serum concentration of 25(OH)D led to a 5% (*β*, − 0.05; 95% CI, − 0.1 to − 0.01, *P* = 0.05107) decrease in the TG, Model I or a 6% (*β*, − 0.06; 95% CI, − 0.11 to − 0.01, *P* = 0.02954) decrease in the TG in Model II. Furthermore, this research estimated that this increase in the concentration of serum 25(OH)D was linked with a 4% (*β*, 0.04; 95% CI, 0.03–0.06, *P* < 0.00001) increase in the HDL in Model I or a 4% (*β*, 0.04; 95% CI, 0.02–0.06, *P* < 0.00001) increase in the HDL in Model II.

**Table 3:** Independent relationship between serum 25(OH)D level and lipids in different models

	Crude Model^a^		Model I^b^		Model II^c^	
	*β* (95% CI)	*P* value	*β* (95% CI)	*P* value	*β* (95% CI)	*P* value
TC	**0.003 (− 0.002, 0.009)**	0.25	**0.003 (− 0.003, 0.009)**	0.30	**0.003 (− 0.003, 0.008)**	0.37
TG	**− 0.005 (− 0.010, − 0.000)**	0.05	**− 0.005 (− 0.010, 0.000)**	0.05	**− 0.006 (− 0.011, − 0.001)**	0.03
HDL	**0.005 (0.003, 0.006)**	< 0.01	**0.004 (0.003, 0.006)**	< 0.01	**0.004 (0.002, 0.006)**	< 0.01
LDL	**− 0.001 (− 0.005, 0.004)**	0.78	**− 0.001 (− 0.006, 0.003)**	0.54	**− 0.001 (− 0.006, 0.003)**	0.55
APO**-**A	**0.004 (0.003, 0.006)**	< 0.01	**0.004 (0.003, 0.006)**	< 0.01	**0.004 (0.002, 0.005)**	< 0.01
LPa	0.80 (− 0.13, 1.74)	0.09	1.14 (0.160, 2.110)	0.02	1.33 (0.34, 2.32)	< 0.01
HCY	0.01 (− 0.02, 0.05)	0.50	− 0.02 (− 0.05, 0.01)	0.23	− 0.01 (− 0.05, 0.02)	0.52

Bolded data are now presented with three decimal places, which improves the sensitivity of the data

*CI* confidence interval, *25(OH)D* 25-hydroxy vitamin D, *TC* total cholesterol, *TG* triglyceride, *HDL* high-density lipoprotein, *LDL* low-density lipoprotein, *APO-A* apolipoproteina, *LPa* lipoproteina, *HCY* homocysteine, *BMI* body mass index, *β-CTX* beta-C-terminal telopeptide of type I collagen, *P1NP* procollagen type I N-terminal propeptide

^a^No adjustment

^b^Adjusted for sex; age of blood collection; year of blood collection; calcium; BMI; season; main diagnosis

^c^Adjusted for Model I plus hypertension; diabetes; β-CTX; P1NP

### Threshold analysis and spline smoothing plot

**Fig. 2:**
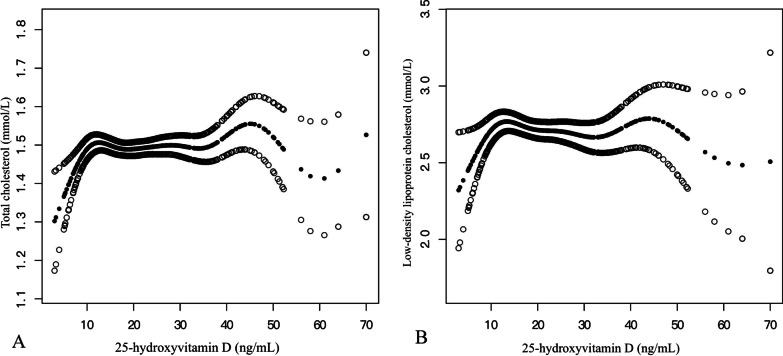
Adjusted smoothed curves corresponding to the relationship between 25(OH)D levels and TC (**A**), LDL-C (**B**). A generalized additive model revealed a threshold nonlinear relationship between 25(OH)D and TC, LDL-C in OP patients. The upper and lower curves represent the range of the 95% confidence interval, and the middle curve represents the correlation between 25(OH)D and TC, LDL-C. Models were adjusted for Sex; Age of blood collection; Year of blood collection; Calcium; BMI; Season; Main diagnosis; Hypertension; Diabetes; β-CTX. The middle curve exhibited an inflection point (*K*) at 10.04 ng/mL. *25(OH)D* 25-hydroxy vitamin D, *TC* total cholesterol, *LDL-C* low-density lipoprotein, *BMI* body mass index, *β-CTX* beta-C-terminal telopeptide of type I collagen

To demonstrate the nonlinear relationship between serum 25(OH)D and blood Lipids, this research utilized threshold effect analysis to accomplish this task. Table 4 displays the results from the threshold effect analysis, which examined the link between the serum concentration of 25(OH)D and the blood lipid profiles of OP patients in the fully adjusted Model II. The *P *value (< 0.05) for LRT indicated a nonlinear relation between 25(OH)D, TC and LDL-C. The two-piecewise linear regression model allowed us to set the turning point (*K*) of 10.04 ng/mL for the serum 25(OH)D concentration to obtain an accustomed flattened curve. Specifically, an expressive, positive association between the blood serum 25(OH)D levels and TC among the studied individuals was detected when 25(OH)D concentration ranged from 0 to 10.04 ng/mL (*β*, 0.107; 95% CI, 0.022–0.192; *P* = 0.014). This research did not detect any association between 25(OH)D and TC when 25(OH)D concentration was > 10.04 ng/mL (*β*, 0.001; 95% CI, − 0.005 to 0.007; *P *value = 0.819). Similarly, a highly positive relationship was proven between LDL-C and 25(OH)D concentrations, when 25(OH)D ranged from 0 to 10.04 ng/mL (*β*, 0.098; 95% CI, 0.03–0.165; *P* = 0.005) and no obvious association was detected between LDL-C and 25(OH)D when the serum levels of 25(OH)D were > 10.04 ng/mL (*β*, − 0.003; 95% CI, − 0.008 to 0.001; *P* = 0.179). Figure 2 shows the relationship between them.

**Table 4:** Threshold effect analysis examining the relationship between 25(OH)D level and lipids

	Model II^a^					
	TC *β* (95% CI) *P* value	TG *β* (95% CI) *P* value	HDL *β *(95% CI) *P* value	LDL *β *(95% CI) *P* value	APO-A *β* (95% CI) *P* value	LPa *β* (95% CI) *P* value
Model A^b^						
One line slope	**0.003 (0.003, 0.008)** 0.37	**− 0.006 (− 0.011, − 0.001)** 0.03	**0.004 (0.002, 0.006)** < 0.01	**− 0.001 (− 0.006, 0.003)** 0.55	0.004 (0.002,0.005) < 0.01	1.33 (0.34,2.32) < 0.01
Model B^c^						
Serum 25(OH)D turning point (K), ng/mL	10.04	10.04	12.87	10.04	24.21	26
< K	0.11 (0.02, 0.19) 0.01	0.05 (− 0.03, 0.12) 0.26	**0.000 (− 0.013, 0.014)** 0.96	0.10 (0.03, 0.17) < 0.01	**0.005 (0.002, 0.008) **< 0.01	2.07 (0.40, 3.75) 0.02
> K	**0.001 (− 0.005, 0.007)** 0.82	**− 0.007 (− 0.012, − 0.001)** 0.01	**0.004 (0.002, 0.006)** < 0.01	**− 0.003 (− 0.008, 0.001)** 0.18	**0.002 (− 0.000, 0.005)** 0.07	0.45 (− 1.44, 2.34) 0.64
Slope 2–Slope 1	− 0.11 (− 0.19, − 0.02) 0.02	− 0.05 (− 0.13, 0.03) 0.20	**0.004** (− 0.01, 0.02) 0.60	− 0.10 (− 0.17, − 0.03) < 0.01	**− 0.003 (− 0.007, 0.002)** 0.23	− 1.62 (− 4.59, 1.35) 0.28
LRT^d^	0.02	0.20	0.59	**0.004**	0.22	0.28

Bolded data are now presented with three decimal places, which improves the sensitivity of the data

*CI* confidence interval, *25(OH)D* 25-hydroxy vitamin D, *TC* total cholesterol, *TG* triglyceride, *HDL* high-density lipoprotein, *LDL* low-density lipoprotein, *APO-A* apolipoproteina, *LPa* lipoproteina, *BMI* body mass index, *β-CTX* beta-C-terminal telopeptide of type I collagen, *P1NP* procollagen type I N-terminal propeptide, *LRT* logarithmic likelihood ratio test

^a^Adjusted for sex; age of blood collection; year of blood collection; calcium; BMI; season; main diagnosis; hypertension; diabetes; β-CTX; P1NP

^b^Linear analysis, *P* value < 0.05 indicates a linear relationship

^c^Nonlinear analysis

^d^*P* value < 0.05 means Model B is significantly different from Model A, which indicates a nonlinear relationship

### Subgroup analysis

This research performed subgroup analyses in the fully adjusted Model II to confirm robust findings. The potential confounders were the patients' age, gender, BMI, primary diagnosis, the season of blood collection, P1NP, Ca, and comorbidities like hypertension, diabetes, year of blood collection, and β-CTX concentrations. All analyses were adjusted for the above eleven covariates except the subgroup variable. Additional file 1: Table S1 shows that all layers were stable.

### Sensitivity analysis

Stratified analyses of both BMI and lipid profiles were conducted using both the WHO [25] and Chinese adult standards [26] for BMI classification. The results of these analyses are presented in Additional file 1: Table S2, showing that significant differences in several lipid biomarkers were observed between different BMI subgroups of the osteoporosis population.

These findings highlight the importance of considering the BMI classification when studying the relationship between osteoporosis and lipid metabolism. Specifically, we found significant differences in lipid biomarkers between the different BMI subgroups established using both the WHO and Chinese adult standards, indicating that the choice of BMI classification may impact the results.

In the TC group, significant differences were observed between osteoporosis patients and those of normal weight range according to the WHO standard, while there were no significant differences between the categories when using the Chinese adult standard. In the HDL group, significant differences were observed between normal-weight and overweight osteoporosis patients using under both the WHO and Chinese adult standards. In the LDL group, a significant difference was observed in the overweight range under both the WHO and Chinese adult standards while in the APO-A group, there were significant differences within the overweight range when using both the WHO and Chinese adult standards. In the LPa group, significant differences were observed between normal, overweight, and obese individuals with osteoporosis when using the WHO standard, while significant differences were only in normal-weight people with osteoporosis when weight was defined according to the Chinese adult standard. In the triglyceride group, a significant difference was found in the overweight range of the osteoporosis population under both the WHO and Chinese adult standards.

Overall, these findings suggest that the BMI classification may be an important factor to consider when studying the relationship between osteoporosis and lipid metabolism. The choice of BMI classification may impact the results, and further research is needed to confirm these findings and explore the potential mechanisms underlying the observed associations.

## Discussion

Although previous cross-sectional studies have investigated the association between osteoporosis and lipid profiles, with some studies finding that higher total cholesterol and triglyceride levels were associated with an increased risk of osteoporosis, the findings of our study add to the existing literature by demonstrating an independent association between vitamin D levels and blood lipid profiles in osteoporosis patients [27]. Our study is the first epidemic Chinese investigation that studied the independent association between the blood serum levels of 25(OH)D and the blood lipid profiles of OP individuals. This research has revealed that in the studied OP patients, the blood serum levels of 25(OH)D were inversely linked with the attention of TG. In contrast, 25(OH)D levels were positively linked with the HDL concentrations. Furthermore, the detected association was nonlinear with the cuff-off value of 25(OH)D concentration of 10.04 ng/mL.

Vitamin D plays a vital role in human physiology, though its exact mechanisms of action are yet unknown. Some authors doubt its improving-the-health effect. On the other side, others have data highlighting its positive impact on the immune system, hormonal regulation and cellular proliferation. Moreover, evidence confirms the link between vitamin D and lipid metabolism without solid details, in which different lipid parameters are affected by changes in the blood serum [28, 29]. Vitamin D insufficiency has been proposed to be linked with CVD [30, 31] without knowing the exact mechanisms through which this is realized. Some results show that it exerted a regulatory effect on cardiomyocytes and vascular smooth muscle cells [32]. Other authors showed that the decreased blood concentrations of 25(OH)D stimulated the renin-angiotensin system and increased blood pressure [33]. In addition, vitamin D has anti-inflammatory activities, thus controlling atherogenesis [32, 34, 35].

The abrogated lipid profile expressed with increased LDL-C and TG concentrations and decreased HDL-C levels has been reported as a CVD risk factor, mainly for atherosclerotic CVD [36, 37]. Moreover, data show that it is a risk factor for an ischemic cerebrovascular stroke [38]. Another risk factor for the pathologies mentioned above is insulin resistance, which is linked with metabolic syndrome [39]. The reason for this is the fact that insulin resistance leads to elevated concentrations of plasma LDL-C and TG and a reduced amount of HDL-C [40]. The results from our study upgrade the above-reported other authors' results with the received data for OP individuals. This research found a positive association between the blood serum concentrations of HDL-C and 25(OH)D, APO-A, and LPA levels in all participants after regulating significant confounders, including age and BMI. In addition, this research established an inverse link between 25(OH)D concentrations and TG levels in all participants. Interestingly, this research found a nonlinear relationship between serum levels of vitamin D, TC and LDL-C. Particularly, when the concentration of 25(OH)D was less than 10.04 ug/mL, they were positively correlated, while it was above 10.04 ug/mL, there was no correlation. These data are unique as they are not reported in previous studies.

Interestingly, our study revealed linear associations between the levels of vitamin D, TC, and LDL-C. When levels of 25(OH)D were less than 10.04 ng/mL, these variables were positively correlated, whereas no such correlations were evident when these concentrations rose above 10.04 ng/mL. These results are also unique, as no one has published them before. Jungert et al. [41] established that 25(OH)D concentrations were negatively related to TC and LDL-C in old German females. The exact correlation was estimated for middle-aged men from Finland [42]. J-m Wang et al. also found that vitamin D serum levels were adversely linked with TC and LDL-C in diabetic individuals [43]. These findings are different from our results. The causes may be the inconsistencies in this study population as it did not include OP patients from China. Although the age group was similar to our cohort of patients, the baseline levels of vitamin D were less in the Chinese population, thus leading to different results.

This research found an inverse link between TG and vitamin D in people with OP, similar to previous studies. Jungert et al. [41] found that 25(OH)D levels were negatively linked with TG among older German women. Karhapää et al. [42] revealed that 25(OH)D serum levels are negatively linked with TG in middle-aged Finnish males. Other authors' results presented that vitamin D concentrations above 10 nmol/l were linked with a decrease in TG (0.52%) among Danish grownups [44]. The same results were found in middle-aged Chinese individuals [45]. Mohammad Ali Arif et al. reported that vitamin D deficiency led to a severe drop in blood serum concentrations of LDL-C, TG and TC. These individuals displayed the highest LDL-C, TG and TC levels, whereas those with mild deficiency had lower levels of the lipids above [46].

Our results showed a positive link between 25(OH)D and HDL concentration in OP individuals. This is in unison with the results of Wang et al. [47], proving that low HDL levels were linked with low vitamin D amounts in Saudi males after correcting senility, BMI, smoking, and physical movement. Other authors also proved that the serum level of 25(OH)D concentration was positively linked with HDL-C without considering factors of obesity in middle-aged men and women [48, 49]. All the above-discussed data prove the link between vitamin D and the blood serum lipid profile in different populations. This suggests that this vitamin has a favorable result on the blood lipid profile. These data, though, need further confirmation.

Preceding reports suggested that the increased calcium absorption in the intestine decreased the synthesis and secretion of hepatic TG [50]. Vitamin D inhibits these processes by activating calcium uptake in the intestines. Data show that the elevated concentrations of intestinal calcium decreased the uptake of fatty acids in the intestines. Moreover, it was proven that the serum levels of LDL-C reduced fat absorption, predominantly saturated fats [51]. Furthermore, calcium promotes the change of cholesterol into bile acids, thus reducing the concentrations of blood serum cholesterol [52]. The elevated concentrations of the parathyroid hormone (PTH) led to an increase in TG, while 25(OH)D suppressed the levels of blood serum PTH [53, 54]. These data prove that vitamin D could affect TG concentrations by modifying the PTH levels. The solid indication that vitamin D shortage was linked with impaired b-cell function and insulin resistance has been demonstrated, thus further showing that this affected lipoprotein metabolism and reduced TG and HDL-C levels [55–57]. Furthermore, vitamin D was proven to be involved in bile acid synthesis in the liver [58]. This proves the potential direct link between it and patients' lipid profile and metabolism.

There are data that low vitamin D levels increase cholesterol. Some authors linked the increased calcium absorption as a risk factor that decreased TG synthesis and secretion in the liver [51]. This proves that insufficient vitamin D concentrations may control these processes. Other authors suggest that insoluble calcium–fatty acid complexes are shaped and constrain fatty acids' intestinal absorption—the last results in cholesterol reduction [59]. A link between the parathyroid hormone, TG and vitamin D has been proposed, in which significant amounts of PTH were linked with enhanced TG and low vitamin D concentrations.

Moreover, data show that increased vitamin D concentrations decreased serum PTH concentrations [47]. Also, there is a strong indication that the lack of vitamin D influences the function of beta-cells, thus causing insulin resistance, lipoprotein metabolism disruption, and ultimately elevated TG and reduced HDL cholesterol concentrations [55]. Hereafter, various types of machinery are most likely to act concurrently and link vitamin D shortage with abrogated lipid profiles.

Specific data link the elevated TG concentrations with overweight. Overweight people lack vitamin D because of the high quantity of subcutaneous fat [60]. Dyslipidemia is also connected with high blood sugar levels, leading to decreased vitamin D concentrations [61]. Physical activity recovers HDL cholesterol, and it facilitates vitamin D concentrations. This research proposes that a vigorous routine including consistent bodily movement may not only support refining dyslipidemia but can also prevent vitamin D deficiency.

This research is the first epidemic Chinese investigation that studied the independent association between the blood serum levels of 25(OH)D and the blood lipid profiles of OP individuals. Our study might have some direct implications for clinical practice. First, supplementation to a serum 25(OH)D concentration of 10.04 ng/mL may benefit TC and LDL-C concentrations in vitamin D-deficient OP patients, although no such benefit is expected for higher 25(OH)D concentrations. Secondly, vitamin D supplementation not only improved bone and muscular health in OP patients [62], but also negatively regulated TG levels and positively regulated HDL, apolipoprotein-A, and lipoprotein A levels, thereby reducing the risk of cardiovascular and cerebrovascular diseases. Third, it helped manage the blood lipid levels in OP patients, especially those with cardiovascular and cerebrovascular diseases. Finally, this research found helpful information for formulating relevant medical guidelines from an evidence-based perspective.

The study has some limitations. First, this study was a retrospective cross-sectional study, so the associations between blood lipids and 25(OH)D do not represent a causal relationship. Furthermore, meaningful endpoint events, such as the occurrence of CVD, were not used as dependent variables in these analyses. Future prospective cohort studies should be conducted based on our study. Next, some significant parameters, including the parathyroid hormone (PTH) levels, cardiac functions, and dietary habits, were not examined. Future studies should thus incorporate these parameters. Third, this research used a single-center design with comparatively minor people numbers; thus, the results could not be generalized to other ethnic groups. Therefore, this study highlights the need for additional research encompassing extensive analyses that include additional biochemical indicators, multi-center RCTs, and people of different ethnicities to better ensure the reliability of these study results.

## Conclusions

Our results demonstrated that the blood serum concentrations of 25(OH)D were negatively correlated with TG levels and positively correlated with HDL, APO-A, and LPA levels in OP patients. In particular, a nonlinear relationship between 25(OH)D levels and concentrations of TC and LDL-C was detected, with positive associations between serum 25(OH)D levels and TC and LDL-C when 25(OH)D concentrations ranged from 0 to 10.04 ng/mL. However, this relationship was not present when 25(OH)D levels were higher than 10.04 ng/mL. Therefore, in the context of the clinical diagnosis and treatment of OP patients, these findings suggest that vitamin D not only has beneficial effects on bone health, but also on blood lipid levels, potentially providing some benefit as a means of preventing CVD.

### Supplementary Information

**Additional file 1.** Table S1 and Table S2.

## Data Availability

The data that support the findings of this study are available from the corresponding author upon reasonable request.
