# Supplementary material for: Association of 25-hydroxyvitamin D levels with lipid profiles in osteoporosis patients: a retrospective cross-sectional study
Source: J Orthop Surg Res. 2023 Aug 14;18:597. doi: 10.1186/s13018-023-04079-8 (PMC10424460; doi:10.1186/s13018-023-04079-8)
Supplement: Supplementary file 1 — Additional file 1. Table S1 and Table S2. [file 13018_2023_4079_MOESM1_ESM.docx]

**Table S1. Subgroup analyses of the relationship of serum 25(OH)D and lipids**

| Sub-group | N | TG  β(95%CI) Pvalue | HDL  β(95%CI) Pvalue | APO-A  β(95%CI) Pvalue | LPa  β(95%CI) Pvalue |
| --- | --- | --- | --- | --- | --- |
| X=25(OH)D |  |  |  |  |  |
| Sex |  |  |  |  |  |
| Male | 333 | -0.001(-0.010,0.008) 0.8589 | 0.007(0.003,0.011) 0.0004 | 0.007 (0.004, 0.010) <0.0001 | 0.927 (-1.064, 2.919) 0.3622 |
| Female | 1730 | -0.005 (-0.010, 0.001) 0.1041 | 0.005 (0.003, 0.007) <0.0001 | 0.005 (0.003, 0.006) <0.0001 | 0.860 (-0.199, 1.918) 0.1116 |
| Age tertile |  |  |  |  |  |
| >50, <=70 years | 1256 | -0.011 (-0.017, -0.004) 0.0014 | 0.005 (0.003, 0.008) <0.0001 | 0.005 (0.003, 0.007) <0.0001 | 1.208 (-0.061, 2.478) 0.0624 |
| >70 years | 807 | 0.001 (-0.006, 0.008) 0.8139 | 0.003 (0.001, 0.006) 0.0199 | 0.004 (0.001, 0.006) 0.0023 | 0.309 (-1.052, 1.669) 0.6567 |
| BMI tertile |  |  |  |  |  |
| <=18.5 kg/m^2^ | 99 | -0.000 (-0.012, 0.012) 0.9634 | 0.008 (-0.003, 0.018) 0.1409 | 0.012 (0.004, 0.020) 0.0051 | -1.644 (-6.543, 3.255) 0.5130 |
| >18.5, <=23.9 kg/m^2^ | 1255 | -0.003 (-0.009, 0.004) 0.3885 | 0.004 (0.001, 0.006) 0.0013 | 0.004 (0.002, 0.006) <0.0001 | 0.712 (-0.453, 1.876) 0.2312 |
| >23.9, <=28 kg/m^2^ | 556 | -0.006 (-0.016, 0.003) 0.1742 | 0.005 (0.002, 0.009) 0.0016 | 0.005 (0.002, 0.008) 0.0018 | 1.623 (-0.155, 3.400) 0.0742 |
| >28, <=35 kg/m^2^ | 147 | -0.021 (-0.046, 0.004) 0.0966 | 0.006 (-0.004, 0.015) 0.2228 | 0.007 (-0.001, 0.015) 0.0991 | -0.931 (-5.782, 3.921) 0.7076 |
| >35 kg/m^2^ | 6 | Null | Null | Null | Null |
| Season of blood collection |  |  |  |  |  |
| Spring (March, April and May) | 503 | 0.004 (-0.005, 0.013) 0.3999 | 0.004 (0.000, 0.008) 0.0306 | 0.004 (0.001, 0.007) 0.0199 | -1.122 (-3.054, 0.810) 0.2556 |
| Summer (June, July and August) | 509 | -0.004 (-0.016, 0.008) 0.5438 | 0.006 (0.002, 0.010) 0.0016 | 0.005 (0.003, 0.008) <0.0001 | 1.305 (-0.333, 2.942) 0.1191 |
| Autumn (September, October and November) | 608 | -0.012 (-0.020, -0.004) 0.0052 | 0.006 (0.002, 0.009) 0.0011 | 0.005 (0.002, 0.008) 0.0015 | 0.607 (-1.108, 2.322) 0.4885 |
| Winter (December, January and February) | 443 | -0.010 (-0.020, -0.001) 0.0369 | 0.005 (0.001, 0.009) 0.0267 | 0.004 (0.000, 0.007) 0.0281 | 2.753 (0.384, 5.122) 0.0234 |
| β-CTX tertile |  |  |  |  |  |
| Low, (0.033-0.233 ng/mL) | 681 | -0.005 (-0.013, 0.002) 0.1680 | 0.005 (0.002, 0.009) 0.0010 | 0.004 (0.002, 0.007) 0.0016 | 0.446 (-0.999, 1.891) 0.5455 |
| Middle,(0.234-0.486 ng/mL) | 680 | -0.006 (-0.017, 0.004) 0.2496 | 0.006 (0.002, 0.009) 0.0005 | 0.004 (0.001, 0.007) 0.0029 | 2.560 (0.926, 4.194) 0.0023 |
| High, (0.487 - 2.26 ng/mL) | 684 | -0.008 (-0.015, -0.000) 0.0443 | 0.001 (-0.003, 0.004) 0.6765 | 0.003 (-0.000, 0.006) 0.0520 | 0.837 (-1.072, 2.746) 0.3904 |
| P1NP tertile |  |  |  |  |  |
| Low, (7 - 32 ug/L) | 653 | -0.007 (-0.015, -0.000) 0.0457 | 0.005 (0.002, 0.009) 0.0008 | 0.005 (0.002, 0.007) 0.0003 | 1.019 (-0.414, 2.453) 0.1641 |
| Middle, (33-55 ug/L) | 709 | -0.000 (-0.011, 0.010) 0.9345 | 0.004 (0.001, 0.007) 0.0157 | 0.004 (0.001, 0.006) 0.0048 | 0.967 (-0.668, 2.602) 0.2469 |
| High, (56-617 ug/L) | 682 | -0.009 (-0.017, -0.000) 0.0503 | 0.002 (-0.002, 0.005) 0.3219 | 0.002 (-0.001, 0.005) 0.2858 | 1.646 (-0.339, 3.631) 0.1047 |
| Ca tertile |  |  |  |  |  |
| Low, (1.24-2.2 mmol/L) | 663 | -0.006 (-0.017, 0.004) 0.2219 | 0.003 (-0.000, 0.006) 0.0588 | 0.003 (0.000, 0.005) 0.0387 | 1.173 (-0.414, 2.759) 0.1481 |
| Middle, (2.21-2.31mmol/L) | 671 | -0.004 (-0.013, 0.005) 0.4213 | 0.005 (0.002, 0.009) 0.0028 | 0.005 (0.002, 0.007) 0.0011 | 1.269 (-0.523, 3.062) 0.1658 |
| High, (2.32-4.98 mmol/L) | 695 | -0.008 (-0.015, -0.001) 0.0243 | 0.004 (0.001, 0.007) 0.0121 | 0.003 (0.001, 0.006) 0.0084 | 0.259 (-1.281, 1.799) 0.7419 |
| Hypertension |  |  |  |  |  |
| No | 1420 | -0.010 (-0.016, -0.004) 0.0010 | 0.006 (0.004, 0.008) <0.0001 | 0.005 (0.004, 0.007) <0.0001 | 0.477 (-0.711, 1.665) 0.4312 |
| Yes | 643 | 0.003 (-0.006, 0.012) 0.4781 | 0.003 (-0.000, 0.006) 0.0867 | 0.003 (0.001, 0.006) 0.0177 | 1.421 (-0.080, 2.921) 0.0640 |
| Diabetes |  |  |  |  |  |
| No | 1772 | -0.005 (-0.010, -0.001) 0.0300 | 0.005 (0.003, 0.007) <0.0001 | 0.005 (0.003, 0.007) <0.0001 | 1.024 (0.029, 2.018) 0.0438 |
| Yes | 291 | -0.003 (-0.019, 0.013) 0.6965 | 0.002 (-0.003, 0.007) 0.4548 | 0.002 (-0.001, 0.006) 0.2070 | -0.352 (-2.921, 2.216) 0.7882 |
| Main diagnosis |  |  |  |  |  |
| OP without fractures | 1391 | -0.005 (-0.010, 0.001) 0.0895 | 0.005 (0.003, 0.007) <0.0001 | 0.004 (0.002, 0.006) <0.0001 | 0.866 (-0.191, 1.922) 0.1085 |
| OPF | 672 | -0.012 (-0.024, 0.001) 0.0762 | 0.003 (-0.001, 0.006) 0.1761 | 0.003 (-0.000, 0.007) 0.0518 | 1.685 (-0.438, 3.807) 0.1205 |
| Year of blood collection |  |  |  |  |  |
| 2015 | 21 | -0.008 (-0.039, 0.023) 0.6131 | 0.006 (-0.001, 0.013) 0.1036 | 0.002 (-0.001, 0.006) 0.2456 | 4.525 (0.213, 8.837) 0.0545 |
| 2016 | 32 | 0.013 (-0.017, 0.043) 0.4182 | -0.005 (-0.017, 0.006) 0.3485 | 0.003 (-0.004, 0.010) 0.4189 | -3.187 (-8.939, 2.565) 0.2865 |
| 2017 | 48 | 0.026 (-0.001, 0.054) 0.0661 | -0.002 (-0.015, 0.011) 0.7857 | 0.002 (-0.006, 0.011) 0.5957 | -1.857 (-7.829, 4.116) 0.5455 |
| 2018 | 128 | 0.004 (-0.012, 0.021) 0.6033 | 0.004 (-0.003, 0.011) 0.2997 | 0.003 (-0.003, 0.010) 0.3545 | 2.684 (-0.709, 6.076) 0.1236 |
| 2019 | 458 | -0.010 (-0.022, 0.001) 0.0857 | 0.003 (-0.002, 0.007) 0.2282 | 0.000 (-0.003, 0.004) 0.8492 | 3.947 (1.610, 6.285) 0.0010 |
| 2020 | 578 | -0.005 (-0.015, 0.005) 0.3422 | 0.006 (0.002, 0.009) 0.0007 | 0.007 (0.004, 0.010) <0.0001 | -1.024 (-2.724, 0.677) 0.2386 |
| 2021 | 705 | -0.007 (-0.015, 0.001) 0.0903 | 0.005 (0.002, 0.009) 0.0037 | 0.005 (0.002, 0.008) 0.0007 | -0.327 (-1.765, 1.111) 0.6558 |
| 2022 | 93 | -0.011 (-0.027, 0.005) 0.1955 | 0.007 (-0.000, 0.014) 0.0626 | 0.007 (0.001, 0.012) 0.0243 | 3.226 (-2.331, 8.782) 0.2600 |

Abbreviations: CI, confidence interval; 25(OH)D, 25-hydroxy vitamin D; TG, Triglyceride; HDL, High-density lipoprotein; APO-A, Apolipoproteina; LPa, Lipoproteina; Ca, calcium; BMI, body mass index; β-CTX, beta-C-terminal telopeptide of type I collagen; P1NP, procollagen type I N-terminal propeptide; OP, osteoporosis; OPF, osteoporotic fracture

**Table S2. Subgroup analyses of the relationship of serum 25(OH)D and lipids about BMI**

| Sub-group | | N | TC  β(95%CI)  *P*-value | HDL  β(95%CI)  *P*-value | LDL  β(95%CI)  *P*-value | APO-A  β(95%CI)  *P*-value | LPa  β(95%CI)  *P*-value | TG  β(95%CI)  *P*-value |
| --- | --- | --- | --- | --- | --- | --- | --- | --- |
| X=25(OH)D |  |  |  |  |  |  |  |  |
| BMI (WHO) |  |  |  |  |  |  |  |  |
| <18.5 kg/m^2^ | 99 | 0.012 (-0.008, 0.032) 0.2369 | -0.000 (-0.013, 0.012) 0.9425 | 0.009 (0.000, 0.018)  0.0550 | 0.001 (-0.009, 0.011) 0.8030 | 1.213 (-5.586, 8.012) 0.7280 | -0.015 (-0.03, -0.000) 0.0518 |  |
| >=18.5, <24.9 kg/m^2^ | 1430 | 0.003 (0.000, 0.007)  **0.0365** | 0.004 (0.002, 0.006)  **0.0003** | -0.001 (-0.002, 0.001) 0.3986 | 0.003 (0.002, 0.005)  **0.0001** | 1.225 (0.093, 2.358)  **0.0342** | -0.004 (-0.010, 0.002) 0.2125 |  |
| >=24.9, <29.9 kg/m^2^ | 472 | 0.000 (-0.007, 0.007) 0.9077 | 0.007 (0.003, 0.011)  **0.0013** | -0.006 (-0.01, -0.002) **0.0024** | 0.005 (0.001, 0.008)  **0.0090** | 3.409 (1.128, 5.690)  **0.0036** | -0.020 (-0.03, -0.008) **0.0014** |  |
| >=29.9 kg/m^2^ | 62 | -0.012 (-0.039, 0.015) 0.4016 | -0.010 (-0.027, 0.008) 0.2836 | 0.003 (-0.009, 0.015) 0.6191 | -0.005 (-0.020, 0.009) 0.4977 | -12.439 (-22.45, -2.38) **0.0201** | 0.021 (-0.023, 0.065) 0.3591 |  |
| BMI (China) |  |  |  |  |  |  |  |  |
| <18.5 kg/m^2^ | 99 | 0.012 (-0.008, 0.032) 0.2369 | -0.000 (-0.013, 0.012) 0.9425 | 0.009 (0.000, 0.018)  0.0550 | 0.001 (-0.009, 0.011) 0.8030 | 1.213 (-5.586, 8.012) 0.7280 | -0.015 (-0.03, -0.000) 0.0518 |  |
| >=18.5, <23.9 kg/m^2^ | 1255 | 0.003 (-0.000, 0.007) 0.0561 | 0.004 (0.002, 0.006)  **0.0012** | -0.001 (-0.003, 0.001) 0.4723 | 0.003 (0.002, 0.005)  **0.0002** | 1.421 (0.193, 2.650)  **0.0236** | -0.002 (-0.009, 0.004) 0.4572 |  |
| >=23.9, <27.9 kg/m^2^ | 552 | 0.003 (-0.003, 0.009) 0.3129 | 0.007 (0.003, 0.010)  **0.0002** | -0.004 (-0.007, -0.001) **0.0188** | 0.005 (0.002, 0.008)  **0.0013** | 1.729 (-0.158, 3.617) 0.0733 | -0.015 (-0.02, -0.005) **0.0033** |  |
| >=27.9 kg/m^2^ | 157 | -0.004 (-0.020, 0.012) 0.6185 | 0.005 (-0.005, 0.015) 0.2971 | -0.006 (-0.015, 0.002) 0.1549 | 0.004 (-0.004, 0.012) 0.3731 | -1.838 (-7.065, 3.389) 0.4922 | -0.023 (-0.050, 0.004) 0.1011 |  |

Adjusted for Sex; Age of blood collection; Year of blood collection; Calcium; Season; Main diagnosis;

Abbreviations: CI, confidence interval; 25(OH)D, 25-hydroxy vitamin D; TC, Total cholesterol; TG, Triglyceride; HDL, High-density lipoprotein; LDL, Low-density lipoprotein; APO-A, Apolipoproteina; LPa, Lipoproteina; BMI, body mass index;
